# Assessment of risky sexual behaviors and risk perception among youths in Western Ethiopia: the influences of family and peers: a comparative cross-sectional study

**DOI:** 10.1186/1471-2458-14-301

**Published:** 2014-04-01

**Authors:** Elias Legesse Negeri

**Affiliations:** 1 College of Medical and Health Sciences, Wollega University, Nekemte, Ethiopia

**Keywords:** Sexual risk behaviors, Risk perception, Out-of-school, In-school, Youths, Family influence, Peer influence, Nekemte

## Abstract

**Background:**

Ethiopia is a developing country with a demographic profile dominated by young population with in the ages of 15–24, constituting one third of the total population. Only little has been explored about the role of parenting process and peers in protecting youths from risky sexual behaviors. Thus, this study tried to assess risky sexual behaviors, risk perception and the influences of family and peers for possible interventions among youths in western Ethiopia.

**Methods:**

The study applied a comparative cross-sectional design triangulated with qualitative study. A pre-tested, structured, interviewer administered questionnaire was used to gather data. SPSS software version 20 was used to perform descriptive statistics, univariate, bivariate and multivariable logistic regression analyses.

**Results:**

Over one third of in-school and 41.4% out-of-school youths reported unprotected sex during the 12 months period prior to interview. More than one third of in-school youths (37.1%) reported to have two and more than two lifetime sexual partners compared to 32.6% of out-of-school youths. Out-of-school youths feel that they are at higher risk of getting HIV than in-school youths (AOR = 2.93; 95% CI: 1.45, 4.35). Youths who had high family connectedness were less likely to commence sexual activity and have multiple sexual partners than their counterparts (AOR = 1.98; 95% CI: 0.63, 0.94) and (AOR = 2.79; 95% CI: 1.24, 4.43) respectively. Having pressure from peer to have sex was significantly associated with having multiple sexual partners (AOR = 2.82; 95% CI: 1.62, 2.49).

**Conclusion:**

A substantial proportion of out-of-school youths engaged in risky sexual behaviors than in-school youths. Parents and peers play a role in shaping the behavior of youths. Consequently, the dimension of good parental process and positive peer factors has to be strengthened.

## Background

In the three decades, that have passed since the world witnessed the onset of the AIDS epidemic, HIV/AIDS has grown into a pandemic that has devastated families, communities and nations worldwide. Globally, the HIV/AIDS epidemic remains a major public health, social, economic and development challenge [1].

Worldwide, risky behaviors related to sexual practices in young people have occupied much of the attention [2]. Studies have reported risky sexual behaviors as a common practice among young people in Sub-Saharan Africa (SSA). Young people in this region frequently were engaged in pre-marital sexual intercourse, with consequences such as unplanned pregnancy [3], Sexually Transmitted Infections (STIs) [4], and HIV/AIDS [5,6]. Against the prevailing cultural norms in Sub-Saharan Africa, such young people also tend to engage in having multiple sexual partners [4-6], concurrent sexual partners [7] and unprotected sexual intercourse [3-6].

Ethiopia is a developing country with a demographic profile dominated by young population with in the ages of 15–24, constituting one third of the total population. No doubt that the HIV/AIDS epidemic has assumed a major public health challenge in Ethiopia [7]. The researchers have shown that there are environments which provide fertile grounds for high sexual risk behaviors and offers great opportunity for HIV risk behavior including unsafe sex [8].

Traditionally, parents have been viewed as having a primary influence on young peoples' sexual behaviors. Researchers consistently find that parent and child connectedness, parental supervision or regulation of children's activities; parents' values against young person intercourse and positive peer behavior decrease the Sexual and Reproductive Health (SRH) problems in young people. So, researches focusing on family and peers influence as a key proximal determinant are a useful focus for potential interventions [9].

Communication between parents and their children on SRH matters promotes safer sexual practices among young people. A number of studies have found that perceptions of peers’ sexual attitudes and behaviors predict sexual risky behavior for young people [10]. Parental monitoring may reduce risky sexual behaviors among young people in Ethiopia, as in other countries [11,12]. In contrast, poor parental monitoring may increase the influence of deviant peers in young people’s lives [13].

To the investigator’s best knowledge, parental and peer influences are among the key mediator for both risk and protective factors that directly impacting young people behaviors and it is necessary to understand the communication gaps, how families and peers influences the sexual behavior of young people, and their own risk perception.

In Ethiopia including the study area, the influences of families and peers on sexual risk behavior and risk perception of youths are not well addressed. Thus, this research was conducted with the aim of assessing the influence of parents and peers on the sexual risky behavior and to determine unsafe sexual practices among in and out-of-school youths in western Ethiopia.

## Methods

### Study design, area and participants

The study was applied a comparative cross-sectional quantitative study design, supplemented by qualitative study, to assess the level and compare the extent of sexual risk behavior, risk perception about HIV/AIDS and the influences made by family and peer on in and out-of-school youths, from March to August 2011.

The study was conducted in western Ethiopia. Out-of-school youths of age 15–24 years, not attending any school in the day or night, unemployed totally or in any formal employment, residing in the study area at least for one year and not married were included. Whereas, in-school youths of age 15–24 years, attending high school, TVET School in the day and not married were included in the study.

### Sample size

Sample size for the quantitative survey was computed using a formula of calculating the difference between two population proportions, with 95% confidence level and 80% power to approximate an acceptable population parameter. Sample size was calculated considering the proportion of youths who were sexually experienced, which is assumed to be the most important risk factor in Ethiopia, was taken as high risk behavior indicator.

The finding of a recent study carried out in eastern and western Ethiopia was taken to represent the proportion of in and out-of-school youths respectively. A study conducted in eastern Ethiopia has found out that of sexually active respondents, 24.8% of in-school youths were sexually experienced [14], and 49.6% of the out-of-school youths were sexually experienced in western Ethiopia [15]. The final sample size was calculated by considering 10% non-response rate, 1.5 design effects, the total sample size was 1,200 youths (600 in-school and 600 out-of-school youths).

### Sampling techniques

For in-school youths: prior to the study, identification of the details of number of schools, classes and sections was conducted. Then, list of students of grade 9^th^ to 12^th^ was prepared from the students’ registration book of each school, class and section (sampling frame). The selection of study participants was based on probability proportional to size to represent each class, section and sex.

Simple random sampling method was used to select the participants by considering male to female proportion (68% males and 32% females). The names of youths corresponding to the selected random numbers were included in the study. Eight in depth interviews were conducted with school directors, gender office, anti-AIDS movement club leaders and members, teachers and for Focus Group Discussions (FGDs), 20 students (in four groups) were selected (selection of the students was purposive to represent the two sexes, grades and active participants of school anti-AIDS movement clubs and different affairs at the school level).

For out-of-school youths, all households which were found in each kebele were initially mapped and numbered, the study subjects were recruited using probability proportional to the number of households in all kebeles, which corresponds to the study units. Every K^th^ of pre-numbered households were visited, until the required numbers of youths were identified for interview in each kebele using systematic random sampling. In case, when more than one eligible respondent is present in a given household, one of them was selected at random to participate in the study and if the respondent was not found at home at the time of data collection appointments was made to come back for interview. Every interviewer had a short checklist to determine appropriateness of study subject for the interview. For FGDs, youths were selected purposively by the principal investigator and data collectors i.e. interviewers of the quantitative survey from the respective kebeles in the town.

### Data collection and processing

A quantitative data was collected using a standardized pre-tested interviewer administered questionnaire adapted from Sexual and Reproductive Health (SRH) questionnaires of World Health Organization (WHO) [16]. The questionnaire was prepared originally in English and then was translated to local language, Afan Oromo and was used to collect data after being pre-tested in schools and towns outside the study area. Data collectors were given three days intensive training. FGDs were made in quite rooms and female youths FGDs were moderated by female moderator (Senior Public Health Nurse) while, that of males’ group was moderated by the principal investigator.

### Data analysis and quality management

Data obtained from the questionnaire was entered, cleaned and prepared for tabulation using statistical data analysis (using SPSS software version 18 and Epi info version 3.5 for windows) techniques. Frequencies for all variables were counted and cross tabulated using percentages. Bivariate logistic regression analysis was used to test possible association of the independent variables with the dependent one. Furthermore, multivariate logistic regression analysis was used to see the net effects of each of the independent variables in explaining variation in the outcome variables. Five percent of the data was double-entered in order to compare and assure the quality of the data.

The FGDs were tape recorded by trained research assistants and took note of all discussions and transcribe for retrieval of the information. Qualitative data were analyzed by organizing the topics raised at the time of group discussion independently i.e. thematic approach and by using the predetermined topics and supplementary suggestions from the participants. The information obtained from the qualitative part was triangulated with the quantitative findings as needed. As much as possible the bias of researchers in the conduct of the study and results was minimized by using observation checklist that should be followed. In addition to this, the qualitative component of this study adheres to the RATS guidelines (Relevance of study question, Appropriateness of qualitative method, Transparency of procedures, Soundness of interpretive approach) for reporting qualitative studies.

### Measurements

#### Sexual risk behavior

It was defined as: unprotected sex (inconsistent use of condoms), having multiple sexual partner, starting sex before age of 18 years and sex with commercial sex workers.

#### Substance use

Use of at least any one of the following substances: alcohol, khat cigarette, shisha, hashish or drug that are assumed to affect level of thinking and increase risk of involving in risky sexual behavior.

#### Consistent condom use

Using condom during or at every sexual encounter.

#### HIV risk perception

Students’ attitude towards perceiving themselves as susceptible to HIV infection.

#### Parental monitoring

Parental monitoring was assessed using a six-item Silverberg’s parental monitoring scale [17]. Items were scored from 1 (never) to 5 (always). Those who scored lower than the median value was considered as low parental monitoring.

#### Parental communication

It was measured with a five-item parent-adolescent communication scale [18]. Items were scored from 1 (never) to 4 (often). Those who scored lower than the median value was considered as low parental communication.

#### Out-of-school youths

Youths within the age range of 15–24 years who were not engaged in any formal education and other vocational trainings (i.e., those dropouts or were not going to school) and who have completed their secondary education, but have not been engaged in any formal employment.

### Ethical consideration

Ethical clearance was obtained from Wollega University, College of Medical and Health Sciences and the research was done in conformity with the ethical guidelines approved by the Institutional Review Board (IRB) of Wollega University. Supporting letter was written by Wollega University to concerned institutions to get institutional consent and official permission. All the information obtained from the respondent was remained anonymous and confidential. Informed consent and/or assent were obtained from each participant both in quantitative and qualitative part.

## Results

### Descriptive findings

The socio-demographic features of the study subjects are shown in Table 1. Out of 1,200 youths, a total of 600 in-school and 583 out-of-school youths participated in the study making the response rate of 98.5%. Nearly one third (32.4%) of females and 67.6% males were participated in the study. Among total (38.3%) of youths were between the age ranges of 15 to 19 years, 61.7% of them were between 20 to 24 years. The mean age was 17.3 (±2.4) and 20.8 (±1.9) years for in and out-of-school youths respectively. More than half, 53.2% of the respondents were protestant and 82.7% youths were Oromo by ethnicity.

**Table 1:** Percentage distribution of socio-demographic characteristics of in and out-of-school youths in Nekemte Town, Western Ethiopia, 2012

**Characteristics**	**Category**	**Number (percentage)**		
		**In-school (n = 600)**	**Out-of-school (n = 583)**	**Total (n = 1183)**
Sex	Male	372 (62%)	411 (70.5%)	783 (66.1%)
	Female	228 (38%)	172 (29.5%)	400 (33.8%)
Age	15-19 years	468 (78%)	104 (22%)	572 (48.3%)
	20-24 years	132 (22%)	479 (72%)	611 (51.6%)
	Mean age (±SD)	17.3 (±3.6)	21.6 (±2.3)	
Religion	Protestant	327 (54.6%)	306 (52.6%)	633 (53.5%)
	Orthodox	204 (36.2%)	212 (36.4%)	416 (35.1%)
	Muslim	55 (9.2%)	46 (7.8%)	101 (8.5%)
	Others	13 (2.2%)	19 (3.2%)	32 (2.7%)
Religiosity	Never attend	20 (3.4%)	30 (5.2%)	50 (4.2%)
	Attend frequently	416 (69.3%)	360 (61.8%)	776 (65.6%)
	Occasionally	164 (27.3%)	193 (32.0%)	357 (30.1%)
Ethnicity	Oromo	489 (81.6%)	486 (83.4%)	975 (82.4%)
	Amhara	76 (12.6%)	87 (15%)	163 (13.8%)
	Guragehe	24 (4.0%)	20 (3.4%)	44 (3.7%)
	Others	11 (1.8%)	7 (1.2%)	18 (1.5%)
Educational status	Not literate	NA	NA	NA
	Elementary	NA	164 (28.2%)	164 (13.8%)
	Secondary or above	600 (100%)	419 (71.8)	1019 (86.1%)
Perceived family economic status	Poor	92 (15.3%)	93 (15.9%)	185 (15.6%)
	Medium	427 (71.1%)	410 (70.4%)	837 (70.7%)
	Rich	81 (13.6%)	80 (14.1%)	161 (13.6%)
Living arrangement	Alone	78 (13%)	104 (18%)	182 (15.3%)
	Both parents	208 (34.8%)	125 (21.4%)	333 (28.1%)
	Single parent	121 (20.1%)	36 (6.0%)	157 (13.2%)
	Relatives	73 (12.1%)	181 (31.1%)	254 (21.4%)
	Friends	120 (20%)	137 (23.5%)	257 (21.7%)
Membership of anti-AIDS movement club	Club members	204 (34.0%)	168 (28.8%)	372 (31.4%)
	Non-club members	396 (66.0%)	415 (72.2%)	811 (68.6%)

*NA*: Not Applicable.

### Sexual behavior

Socio-demographic and behavioral correlates of sexual activity of the study participants’ are shown in Table 2. From the total respondents, 212 (35.3%) of in-school and 241 (41.4%) out-of-school youths had sex. Disaggregated by sex, 150 (40.3%) of males had had sex compared to 62 (27.2%) of females from in-school youths. While, 179 (43.5%) of males had sex compared to 62 (36%) of females of out-of-school youths. The mean age of sexual initiation was 18.01 (±2.27) and 18.72 (±3.41) years overall for in and out-of-school youths respectively. Females had more likely started sexual activity earlier than males in both groups which was statistically significant (P value < 0.05).

**Table 2:** Socio-demographic and behavioural correlates of sexual activity among in and out-of-school youths in Nekemte Town, Western Ethiopia, 2012

**Characteristics**		**Total**	**Ever had sex (%)**	**Crude OR (95% CI)**	**Adjusted OR (95% CI)**
Schooling status	In-school	600	212 (35.3)	1.00	1.00
	Out-of-school	583	241 (41.3)	3.99 (0.81, 2.47)	1.96 (0.29, 1.21)
Membership anti-AIDS movement club	Club member	372	96 (25.8)	0.82 (1.01, 1.99)	1.45 (0.84, 3.88)
	Non-club member	811	357 (44.0)	1.00	1.00
Peer pressure to have sex	Yes	729	340 (46.6)	1.12 (0.88, 2.51)	1.96 (1.21, 3.04) **
	No	454	113 (24.9)	1.00	1.00
Age group	15-19	572	159 (27.8)	1.00	1.00
	20-24	611	294 (48.1)	0.77 (0.17, 1.43)	0.95 (0.23, 3.96)
Parental monitoring	Lower	592	368 (61.6)	0.89 (0.43, 1.37)	1.48 (0.43, 2.52)
	More	591	85 (14.3)	1.00	1.00
Parent youths discussion about sexual matters	Yes	916	315 (34.3)	1.00	1.00
	No	267	142 (53.1)	1.21 (1.08, 3.31)	2.23 (1.29, 3.96) **
Attending religious institution	Never attend	50	182 (92.0)	0.92 (2.60, 3.52)*	1.66 (1.93, 3.31)**
	Occasionally	357	174 (62.7)	3.57 (1.92, 6.66)*	3.73 (1.57, 8.85)**
	Attend frequently	776	97 (22.4)	1.00	1.00
Best Friend experienced sex	No	472	97 (20.6)	1.00	1.00
	Yes	682	341 (50.0)	1.95 (0.44, 2.42)	1.41 (0.24, 0.91) **
	Don’t Know	29	15 (51.7)	0.47 (0.21, 1.07)	0.40 (0.15, 1.06)
How many of your friends have had sex?	None of them	431	28 (6.5)	1.00	1.00
	Few of them	335	91 (27.1)	2.08 (1.10,3.90)*	1.48 (0.65, 10.81)
	Half of them	210	150 (71.4)	1.66 (0.67, 4.1)	0.68 (0.16, 2.27)
	Most of them	256	184 (71.9)	1.06 (0.54, 2.56)	1.87 (0.53, 3.25)
Living arrangement	Both parents	615	69 (11.2)	1.00	1.00
	Alone	335	236 (70.4)	2.53 (1.34, 4.76)*	1.43 (1.70, 5.92)**
	Single parent	66	37 (56.0)	2.24 (0.76, 6.61)	1.33 (0.37, 4.79)
	Relatives	67	46 (68.6)	2.68 (1.02,7.09)*	0.98 (2.24, 3.99)**
	Friends	106	64 (60.4)	3.09 (1.25,7.60)*	1.04 (3.24, 4.58)**
Ever consumed alcohol	Yes	842	373 (44.2)	2.38 (1.62, 3.49)*	3.41 (2.32, 6.16)**
	No	341	80 (23.5)	1.00	1.00
Chew Khat	Yes	173	130 (75.1)	1.60 (1.13, 2.27)*	1.25 (0.83, 1.89)
	No	1010	323 (31.9)	1.00	1.0

*Significant for CrudeOR **Significant for AdjustedOR.

More than one third, 223 (37.1%) of in-school and 190 (32.6%) of out-of-school youths reported that they had two and more than two lifetime sexual partners, of which 109 (57.4%) and 81 (52.6%) males and females respectively.

Of those who reported having had sexual intercourse, 136 (64.2%) in-school and 172 (71.4%) out-of-school youths had sex in the past 12 months. Amongst sexually active youths, 147 (69.3%) and 143 (59.7%) in and out-of-school youths reported contraceptive use at first sexual intercourse respectively. In contrast, at most recent sexual intercourse, more in-school youths, 343 (57.2%) than out-of-school youths, 282 (48.4%) reported consistent condom use at their most recent sex.

Youths reported attending religious institutions frequently were more likely to reject sexual activity than those who never and occasionally attend religious institutions i.e. the proportion of youths reporting sexual activity differs significantly with respect to religious attachment. Youths who lived with their relatives, with friends, or alone were significantly more likely to report sexual activity than those who lived with both biological parents (AOR = 2.68; 95% CI: 1.02-7.09), (AOR = 3.09; 95% CI: 1.25- 7.60) and (AOR = 3.14; 95% CI: 1.10-9.02) respectively.

### Substance use

The study revealed that 122 (20.3%), 163 (27.2%) of the in-school youths and 228 (39.1%), 238 (40.9%) of the out-of-school youths chewed khat at least once in their lifetime and they were current khat chewers (in the last 3 months) respectively. Youths who had high perceived family connectedness were less likely to report history of khat consumption (P value <= 0.05) and drinking alcohol (P value < 0.05).

Concerning alcohol drinking habits, 250 (41.7%), 183 (30.5%) of in-school youths and 315 (54.1%), 241 (41.6%) of out-of-school youths reported that they drank alcohol at least once in their lifetime and they drank alcohol in the last three months respectively. The odds of having had sex were significantly three times higher for youths drinking alcohol than youths who didn’t drink alcohol (AOR = 3.41; 95% CI: 2.32, 6.16) (Table 2).

In general those respondents who have a family members who drink alcohol, chew khat and smoke cigarette were more likely to drink alcohol, chew khat and smoke cigarette (AOR = 5.12; 95% CI: 3.32, 10.41), (AOR = 4.22; 95% CI: 2.92, 11.12) and (AOR = 3.82; 95% CI: 3.18, 3.96) respectively.

### Risk perception of youths

In the present study, about 204 (34.0%) of in-school and 326 (54.3%) of out-of-school youths thought that they were at risk of HIV infection. Risk perception of HIV significantly related with schooling status and number of sexual partners.

Out-of-school youths feel that they are at high risk than in-school youths (AOR = 2.93; 95% CI: 1.45, 4.35) and youths who were reported to have two or more life time sexual partners perceived themselves as at high risk of getting HIV than those with single sexual partner (AOR = 2.79; 95% CI: 1.24, 4.43) (Table 3).

**Table 3:** Comparison of selected socio-demographic and behavioral variables with youths own risk perception in Nekemte Town, Western Ethiopia, 2012

**Characteristics**		**Total**	**Risk perception (%)**	**Crude OR (95% CI)**	**Adjusted OR (95% CI)**
Membership of anti-AIDS movement club	Club member	372	356 (95.7)	2.60 (1.13, 6.12)*	3.32 (1.28, 7.29) **
	Non-club member	811	274 (33.8)	1.00	1.00
Sex	Male	783	268 (34.2)	0.79 (0.57, 2.43)	1.11 (0.49, 2.03)
	Female	400	362 (90.5)	1.00	1.00
Age group	15-19	572	367 (64.1)	1.64 (1.17, 2.34)*	0.82 (0.38, 1.79)
	20-24	611	263 (45.9)	1.00	1.00
Schooling status	In-school	600	349 (58.1)	1.00	1.00
	Out-of-school	583	281 (48.1)	1.24 (0.16, 9.33)	2.93 (1.45, 4.35) **
Knowledge of HIV prevention	Knowledgeable	895	493 (55.0)	1.30 (1.06, 2.22)*	0.67 (0.10, 4.52)
	Not knowledgeable	288	137 (47.6)	1.00	1.00
Number of life time sexual partner	≥ Two	413	360 (57.2)	0.93 (0.89, 3.54)	2.79 (1.24, 4.43) **
	One	770	270 (42.8)	1.00	1.00
Condom use during any sexual contact	Yes	625	255 (40.8)	1.12 (0.36, 1.22)	2.30 (0.14, 0.67) **
	No	558	375 (67.2)	1.00	1.00
Willingness to get VCT	Yes	704	412 (58.5)	0.85 (0.50, 1.43)	0.67 (0.30, 1.49)
	No	482	218 (45.2)	1.00	1.00
Drink alcohol	Yes	842	372 (44.1)	0.95 (0.57, 1.98)	1.99 (1.54, 4.86) **
	No	341	258 (75.6)	1.00	1.00
Khat chewing	Yes	173	159 (91.9)	1.80 (0.70, 5.94)	1.39 (0.30, 4.28)
	No	1010	471 (46.6)	1.00	1.00

*Significant for CrudeOR **Significant for AdjustedOR.

Those who use condom during any sexual intercourse perceived that they are at lower risk of HIV infection than those who didn’t use condom (AOR = 2.30; 95% CI: 0.14, 0.67) and youths who drank alcohol feel that they are at higher risk of HIV infection than those who were not (AOR = 1.99; 95% CI: 1.54, 4.86). Almost all 356 (95.7%) of anti-AIDS movement club members perceived that they are at risk or may be at risk of acquiring the disease than the non-club members, 363 (44.7%). The difference was statistically significant (AOR = 3.32; 95% CI: 1.28, 7.29) (Table 3).

### Influence of family on sexual risk behavior of youths

#### Perceived youth-family connectedness

The percentage of in-school youths who had high family connectedness was higher than their counterparts’ out-of-school youths (60.0% versus 51.2%). There was statistically significant difference in commencing premarital sex and having multiple sexual partners between respondents who had high and low family connectedness; youths who have had high family connectedness were less likely to commence premarital sexual activity and less likely to had multiple sexual partners than their counterparts (AOR = 1.96; 95% CI: 1.48, 3.5) and (AOR = 2.83; 95% CI: 2.61, 6.73) respectively (Tables 4 and 5).

**Table 4:** Comparison of risk sexual behavior variable (Premarital sex) and some characteristics of parenting process among youths in Nekemte Town, Western Ethiopia, 2012

**Characteristics**		**Had premarital sex**		**Crude OR (95% CI)**	**Adjusted OR (95% CI)**
		**Yes (%)**	**No (%)**		
Parents know whereabouts	Always	152 (33.5)	426 (58.4)	0.71 (0.42, 2.82)	1.33 (0.59, 2.42)
	Sometimes	185 (40.9)	220 (30.1)	1.58 (0.63, 3.12)	2.26 (0.48, 1.94)
	Never	116 (25.6)	84 (11.5)	1.00	1.00
Parents know your close friends	All of them	139 (30.7)	440 (60.3)	1.59 (1.29, 2.44)*	0.88 (0.58, 1.91)
	Some of them	175 (38.6)	200 (27.4)	0.72 (0.42, 2.23)	1.32 (0.52, 3.92)
	Not at all	139 (30.7)	90 (12.3)	1.00	1.00
Parents know what you are doing when you are away from home	Always	173 (38.2)	471 (64.5)	1.04 (0.48, 0.84)	2.92 (1.84, 4.56)**
	Sometimes	183 (40.4)	147 (20.2)	1.24 (0.16, 9.33)	2.93 (0.45, 4.35)
	Never	97 (21.4)	112 (15.3)	1.00	1.00
Parents know your plans for the coming day	Always	243 (53.7)	512 (70.1)	1.33 (0.68, 1.84)	0.83 (0.73, 2.42)
	Sometimes	149 (32.8)	134 (18.3)	0.94 (0.59, 1.63)	0.90 (0.43, 1.87)
	Never	106 (23.5)	85 (11.6)	1.00	1.00
Family connectedness	Low	274 (60.4)	179 (24.5)	1.00	1.00
	High	179 (39.6)	551 (75.5)	1.22 (0.88, 3.45)	1.96 (1.48, 3.58)**
Parents know about your close friends activities and interest	Yes	148 (32.6)	353 (48.4)	1.27 (0.26, 2.23)	2.03 (0.48, 0.87)**
	No	305 (67.4)	377 (51.6)	1.00	1.00

*Significant for CrudeOR **Significant for AdjustedOR.

**Table 5:** Comparison of risk sexual behavior variable (Multiple sexual partnerships) and some characteristics of parenting process among youths in Nekemte Town, Western Ethiopia, 2012

**Characteristics**		**Multiple sexual partnership**		**Crude OR (95% CI)**	**Adjusted OR (95% CI)**
		**Yes**	**No**		
Parents know whereabouts	Always	159 (38.4)	483 (62.8)	0.49 (0.57, 2.43)	1.11 (0.49, 2.03)
	Sometimes	175 (42.3)	216 (28.1)	1.32 (0.95, 3.32)	1.87 (0.87, 4.53)
	Never	80 (19.3)	78 (10.1)	1.00	1.00
Parents know your close friends	All of them	145 (35.2)	504 (65.4)	1.64 (1.17, 2.34)*	0.82 (0.38, 1.79)
	Some of them	166 (40.3)	178 (23.1)	2.56 (0.26, 4.26)	2.01 (0.59, 5.63)
	Not at all	101 (24.5)	89 (11.5)	1.00	1.00
Parents know what you are doing when you are away from home	Always	138 (33.4)	464 (60.3)	1.24 (0.16, 9.33)	2.93 (1.45, 4.35)**
	Sometimes	162 (39.2)	212 (27.5)	1.67 (0.51, 5.41)	2.54 (0.87, 5.33)
	Never	113 (27.4)	94 (12.2)	1.00	1.00
Parents know your plans for the coming day	Always	201 (48.6)	556 (72.2)	1.58 (0.94, 3.89)	2.21 (0.81, 4.52)
	Sometimes	149 (36.1)	120 (15.6)	1.44 (0.88, 2.36)	0.90 (0.44, 1.86)
	Never	63 (15.3)	94 (12.2)	1.00	1.00
Family connectedness	Low	298 (72.2)	164 (21.3)	1.00	1.00
	High	115 (27.8)	606 (78.7)	0.93 (0.89, 3.54)	2.83 (2.61, 6.73)**
Parents know about your close friends activities and interest	Yes	163 (39.4)	440 (57.2)	1.12 (0.36, 1.22)	2.30 (0.14, 0.67)**
	No	250 (60.6)	330 (42.8)	1.00	1.00

*Significant for COR **Significant for AOR.

#### Perceived parental monitoring/ family control

Regarding living arrangements of the parents’ of youths, (61.4%) of in-school and (42.4%) of out-of-school youths currently live together. From the total respondents, 392 (65.3%) of in-school and 271 (46.4%) of out-of-school youths agreed on parental monitoring of youths day-to-day activities. Almost three fourth, 443 (73.8%) of the in-school and 281 (48.2%) perceived that their parents didn’t know their sexual experience.

Hence, from the total youths participated in the study, 223 (37.1%) of in-school and 370 (63.4%) of out-of-school youths were categorized as having less perceived parental monitoring. Youths who reported that their parents always knows what they are doing when they are away from home and those youths who reported that their parents knows every activity and interest of their close friends were less likely to had premarital sex (AOR = 2.92; 95% CI: 1.84, 4.56) and (AOR = 2.03; 95% CI: 0.48, 0.87) respectively (Table 4).

#### Parent-youth communication and discussion

Among the total respondents, only 187 (31.2%) of mothers and 156 (26.7%) of fathers will answer to them helpfully if they asked about Sexual and Reproductive Health (SRH) questions. Generally, 351 (88.6%) in-school and 268 (45.9%) out-of-school youths reported that they had ever discussed SRH or HIV/AIDS. Compared to in-school youths, out-of-school youths were significantly more likely to discuss with their family (AOR = 1.48; 95% CI: 1.82, 4.89). The odds of having had sex were two fold higher among youths who don’t discussed about sexual matters than who discussed (Table 2).

#### Influence of peers on sexual risk behavior of youths

From the total respondents, 59.2% of in-school and 64.1% of out-of-school youths, of which almost three fourth, 72.2% of in-school and 68.6% out-of-school youths were males and reported as they have had pressure from their peer groups to engage in sexual activities.

Youths who had peer pressure to have sexual intercourse and those who had friends already engaged in sexual intercourse were more likely to have sexual experience (AOR = 1.96; 95% CI: 1.21, 3.04) and (AOR = 1.41; 95% CI: 0.24, 0.91) respectively (Table 2).

In general, when we compare youths with respect to living arrangement, youths living with their friends, alone and one biological parent were significantly more likely to report that they have peer pressure to have sexual intercourse as compared to those who live with both biological parents (AOR = 2.31; 95% CI: 1.45, 4.64); (AOR = 2.87; 95% CI: 1.69, 4.67) and (AOR = 2.97; 95% CI: 1.94, 3.96) respectively.

Having female friends who have had sex was significantly associated with premarital sexual activity (AOR = 1.82; 95% CI: 1.38, 4.79). Ever drinking alcohol and schooling status of youths were significantly associated with premarital sexual activity (AOR =1.67; 95% CI: 1.30, 5.49) and (AOR = 2.73; 95% CI: 1.85, 5.33) respectively (Table 6).

**Table 6:** Comparison of sexual risk behavior variable (Premarital sex) and some characteristics of peer influence among youths in Nekemte Town, Western Ethiopia, 2012

**Characteristics**		**Had premarital sex**		**Crude OR (95% CI)**	**Adjusted OR (95% CI)**
		**Yes**	**No**		
Having male friends who have practiced sex	Yes	210 (46.4)	234 (32.1)	0.79 (0.57, 2.43)	1.85 (0.81, 5.31)
	No	242 (53.6)	496 (67.9)	1.00	1.00
Having female friends who have practiced sex	Yes	183 (40.5)	207 (28.3)	1.64 (1.17, 2.34)*	1.82 (1.38, 4.79)**
	No	270 (59.5)	523 (71.7)	1.00	1.00
Schooling status of youths	In-school	212 (35.3)	472 (64.7)	1.24 (0.16, 9.33)	2.73 (1.85, 5.33)**
	Out-of-school	241(41.3)	429 (58.7)	1.00	1.00
Having friends who drink alcohol	Yes	305 (67.4)	273 (37.4)	1.44 (0.88, 2.36)	1.90 (0.54, 4.86)
	No	148 (32.6)	457 (62.6)	1.00	1.00
Having friends who smoke cigarette	Yes	355 (78.4)	156 (21.4)	1.3 (1.06, 2.22)*	1.67 (0.10, 4.52)
	No	98 (21.6)	574 (78.6)	1.00	1.00
Having friends who chew khat	Yes	318 (70.1)	163 (22.3)	0.93 (0.89, 3.54)	0.49 (0.30, 1.49)
	No	135 (29.9)	567 (77.7)	1.00	1.00
Discuss about sexuality with peers	Yes	333 (73.4)	453 (62.1)	1.12 (0.36, 1.22)	2.30 (0.14, 0.67)**
	No	75 (16.6)	277 (37.9)	1.00	1.00
Ever drink alcohol	Yes	386 (85.3)	295 (40.4)	0.85 (0.50, 1.43)	1.67 (1.30, 5.49)**
	No	67 (14.7)	435 (59.6)	1.00	1.00
Ever Khat chew	Yes	392 (86.6)	273 (37.4)	1.80 (0.70, 5.94)	1.39 (0.30, 4.28)
	No	61 (13.4)	457 (62.6)	1.00	1.00

*Significant for CrudeOR **Significant for AdjustedOR.

Youths having male friend who have practiced sex had three fold increased odds of having multiple sexual partners compared to those who don’t have, (AOR = 3.11; 95% CI: 1.60, 8.57). Having pressure from peer to have sex was significantly associated with having multiple sexual partners (AOR = 2.82; 95% CI: 1.62, 3.49) (Table 7).

**Table 7:** Comparison of sexual risk behaviors variable (Multiple sexual partnerships) and some characteristics of peer influence among youths in Nekemte Town, Western Ethiopia, 2012

**Characteristics**		**Multiple sexual partners**		**Crude OR (95% CI)**	**Adjusted OR (95% CI)**
		**Yes**	**No**		
Having male friends who have practiced sex	Yes	326 (79.4)	284 (36.9)	0.79 (0.57, 2.43)	3.11 (1.60, 8.57)**
	No	85 (20.6)	486 (63.1)	1.00	1.00
Peer pressure to have sex	Yes	298 (72.2)	307 (39.9)	1.64 (1.17, 2.34)*	2.82 (1.62, 3.49)**
	No	74 (17.8)	463 (60.1)	1.00	1.00
Schooling status of youths	In-school	223 (37.1)	484 (62.9)	1.00	1.00
	Out-of-school	190 (32.6)	519 (67.4)	1.24 (0.16, 9.33)	2.93 (1.45, 4.35)**
Having friends who drink alcohol	Yes	323 (78.2)	204 (26.5)	1.00	1.00
	No	90 (21.8)	566 (73.5)	1.44 (0.88, 2.36)	2.51 (0.83, 6.33)
Peer pressure to smoke cigarette	Yes	331 (80.2)	172 (22.4)	1.30 (1.06, 2.22)*	2.65 (0.63, 7.58)
	No	82 (19.8)	598 (77.6)	1.00	1.00
Peer pressure to chew khat	Yes	306 (74.1)	226 (29.4)	0.93 (0.89, 3.54)	2.79 (0.24, 4.43)
	No	107 (25.9)	544 (70.6)	1.00	1.00
Peer pressure to drink alcohol	Yes	316 (76.4)	194 (25.2)	1.12 (0.36, 1.22)	2.30 (0.14, 5.67)
	No	97 (23.6)	576 (74.8)	1.00	1.00
Ever drink alcohol	Yes	339 (82.1)	155 (20.1)	0.85 (0.50, 1.43)	1.67 (1.30, 5.49)**
	No	74 (17.9)	615 (79.9)	1.00	1.00
Ever Khat chew	Yes	332 (80.3)	202 (26.2)	1.80 (0.70, 5.94)	1.39 (0.30, 4.28)
	No	81 (19.7)	88 (73.8)	1.00	1.00

*Significant for CrudeOR **Significant for AdjustedOR.

## Discussion

This study attempted to provide some insights on sexual risk behaviors and risk perception of youths about HIV/AIDS. In addition, the study tried to see the influences of parents or families and peers on the sexual risk behavior of youths.

This current study illustrates that as 35.3% of in-school and 41.4% out-of-school youths had sex. This finding is slightly higher than the study done in Butajira 20.2% [19]. In contrast, the figure in this study is slightly low when compared with the results of similar studies in Tanzania which was 54% for both sexes [20].

In this study, females had more likely started sexual activity earlier than males in both groups which were statistically significant. This study finding contradicts with the previous study conducted in eastern Ethiopia [14]. This difference was supported by FGDs result and explained as there are lower values (advantages attached to virginity) were given for virginity at marriage for females and to some extent the condition of adopting cultures of westerns by overlooking the native culture.

Comparatively in this study, 32.6% out-of-school and 37.1% of in-school youths had in excess of two lifetime sexual partners. This finding is considerably greater than the study done in Dares Salaam, 25% of respondents had more than one sexual partner at a time and the mean number of lifetime sexual partners is 2.7, which is in excess of this study [21]. In the same way, participants from the FGDs disapproved premarital sex because of its consequences with diverse problems related to SRH including unwanted pregnancy, STIs and HIV/AIDS.

In the present study, greater than half, 52.7% of the sexually active youths ever used condom and only 42.7% of these claimed to have used condom consistently on their subsequent sexual encounters. In Butajira, of those who experienced sexual intercourse for the first time, only one third reported that they had used condom [19]. The reported low utilization rate of consistent condom use in this study is an indication of the fact that high-risk behaviors are still widely practiced among youths in the study area. This calls for a well-organized information, education and communication through peer educators to bring about behavioral change.

In this study, youths who drink alcohol were three times more likely to engage in sexual activity than those who didn’t drink. This finding is supported by similar study in rural Ethiopian youths and Butajira [19,22,23].

In this study, there is much better risk perception HIV infection than the study conducted in South Gondar, in which, 2.2% of youths thought that they were at risk of HIV infection [24]. Even though there is better level of risk perception in this study, still it needs extraordinary attention to upgrade it in order to prevent youths from HIV/AIDS and SRH problems.

Youths who were reported to have two or more sexual partners, use condom inconsistently and drank alcohol perceived themselves as at high risk of getting HIV than their counterparts. Similarly in study done in Gondar, the lower level of risk perception of HIV/STIs, associated with condom use, knowledge on HIV transmission, number of sexual partners and khat chewing. These figures are also consistent with other similar study [25].

In this study, 72.5% of club members perceived that they are at risk or may be at risk of acquiring the disease than the non-club members 54.7%. The difference was statistically significant and comparable with study done in western Ethiopia [15]. This shows us, it is important to establish and strengthen anti-AIDS club movement in all schools and youth centers, so that they can provide adequate information and services they need for in-school and out-of-school youths on reproductive and sexual issues especially condom provision in a friendly manner.

Youths who had high family connectedness were less likely to commence sexual activity and had multiple sexual partners than their counterparts. Different research elsewhere showed that young adults who have more perceived parental connectedness have a reduced level of risk taking behavior including premarital sex [11]. The result of FGDs also supports as parental monitoring and connectedness has tremendous uses because families do have a significant role advising, leading, communicating with and increasing the relationship existing between them.

Youths who reported that their parents always know what they are doing when they are away from home and those youths who reported that their parents know every activity and interest of their close friends were less likely to had premarital sex. The finding of this study is comparable with a study which also showed a significant effect for the above mentioned measures but, family connectedness which is failed to show association in these researches [26]. In the present study, family connectedness showed an association.

Similar to this study finding, other studies conducted at different parts of Ethiopia found that higher level of perceived parental monitoring was associated with less sexual activities [25,26]. In contrary to this, others found that too much of perceived parental monitoring was associated with higher odds of taking risk among adolescents [27]. Similarly the result from the FGDs in this research showed that; too much parental monitoring will end up with undesirable values on sexual risk behavior of youths. Another FGD result conforms to this study was when the monitoring is forced type it may not bring about positive behavioral changes, even sometimes results in negative outcomes.

Other study also conclude that positive outcome on adolescent sexual behavior may not be expected unless young people’s voluntary disclosure of information about their lives [20]. The result of this study is consistent with studies conducted in different areas which mainly showed the association [25,28,29].

This finding is also consistent with the FGD result, which may indicate that there is a gap in discussing the positive aspect of adolescent sexuality related issues. Thus, if adolescents discussed only a negative outcome about sexuality with their parents, they will be highly unlikely to turn to their parents to discuss sexual matters as they get older. On the other hand, positive communication about sexual information, feelings, attitudes, values and behavior when children are young often leads to ongoing discussions as they mature. Establishing an environment conducive to open and comfortable communication is therefore, extremely important.

Compared to in-school youths, out-of-school youths were significantly more likely to discuss with their family. The main reason forwarded from FGD is that as the age youths grow from adolescence to youth, the connection they had becomes stronger to their peers than families, in addition to this, issues like culture, shame, and fear of families could also be the reason for preferring non-family members.

Moreover, in this study parents’ educational status particularly of mothers’ is positively linked with discussion on different SRH issues, since they can provide reliable information and will increase the knowledge of parents on SRH matters and this will further builds their confidence to talk with their youths about SRH concern which protect them at risk of various RH challenges. This finding was also consistent with study done in USA [12].

This particular study proved that youths who had peer pressure to have sexual intercourse and those who had friends already engaged in sexual intercourse were more likely to have sexual experience. The FGD conducted in similar research in Ethiopia also demonstrates that the repeated exposure of youths to peer groups who have already engaged in risk behavior let them to adapt and perform their activities. Apart from the above idea, FGD participants raised that as there are youths who may have good family monitoring and connectedness will going to resist the pressure from peer groups.

This study showed that having a male friend who have practiced sex and having pressure from peer to have sex were significantly associated with having multiple sexual partner. Another similar research findings also identified that youth-peer communication about sex was associated with more number of sexual partners [10].

The strength of this study is its inclusion of both in school and out-of-school youths, hence it can be generalizable. The response rate for the study was high. Communication on SRH, sexual behaviors and attitude outcomes are sensitive and based on self-reported information, therefore some information may not be reported honestly and the possibility of underestimation cannot be ruled out. Some sort of desirability and recall bias may not be eliminated. The study measures parental monitoring, connectedness and communication on the side of young people’s perception, which may not reflect what parents perceive and actually doing.

## Conclusions

This study has shown that a considerable proportion of out-of-school youths engage in risky sexual behaviors than in-school youths. Good parental monitoring and high parental connectedness are related to better sexual health and peers play a role in shaping the behavior young people, as peers tend to choose those who are similar to themselves. There is a need to equip and educate parents on different SRH issues with appropriate IEC materials and communication skills on sexuality and RH related issues. Encourage and empower parents to start to communicate with their children on sexual matters while the children are still in late childhood or early teenage years, before they become sexually active.

## Competing interests

Author declares that he has no competing interests.

## Author’s contributions

EL has conceived of the study, carried out the overall design and execution of the study, design of questionnaires, performed the data collection, performed the statistical analysis and served as the lead author of the manuscript. The author read and approved the final manuscript.

## Pre-publication history

The pre-publication history for this paper can be accessed here:

http://www.biomedcentral.com/1471-2458/14/301/prepub
